# Locomotor Behavior of Chickens Anticipating Incline Walking

**DOI:** 10.3389/fvets.2017.00233

**Published:** 2018-01-10

**Authors:** Chantal LeBlanc, Bret Tobalske, Bill Szkotnicki, Alexandra Harlander-Matauschek

**Affiliations:** ^1^Department of Animal Biosciences, University of Guelph, Guelph, ON, Canada; ^2^Division of Biological Sciences, University of Montana, Missoula, MT, United States

**Keywords:** incline, walking, locomotion, adaptation, anticipation, ground reaction force, bird

## Abstract

Keel bone damage (KBD) is prevalent in hens raised for egg production, and ramps between different tiers in aviaries have potential to reduce the frequency of falls resulting in KBD. Effective use of ramps requires modulation of locomotion in anticipation of the incline. Inadequate adaptive locomotion may be one explanation why domestic layer hens (*Gallus gallus domesticus*) exhibit high rates of KBD. To improve understanding of the capacity of hens to modulate their locomotion in anticipation of climbing, we measured the effects of incline angle upon the mechanics of the preparatory step before ascending a ramp. Because the energetic challenge of climbing increases with slope, we predicted that as angle of incline increased, birds during foot contact with the ground before starting to climb would increase their peak force and duration of contact and reduce variation in center of pressure (COP) under their foot. We tested 20 female domestic chickens on ramp inclines at slopes of +0°, +40°, and +70° when birds were 17, 21, 26, 31, and 36 weeks of age. There were significantly higher vertical peak ground reaction forces in preparation at the steepest slope, and ground contact time increased significantly with each increase in ramp angle. Effects upon variation in COP were not apparent; likewise, effects of limb length, age, body mass were not significant. Our results reveal that domestic chickens are capable of modulating their locomotion in response to incline angle.

## Introduction

Keel bone damage (KBD) is a critical form of injury in commercial aviaries in which domestic chickens (*Gallus gallus*) are raised for egg production, but the sources of this injury are poorly understood (1). Chickens routinely use various forms of locomotion in aviaries, including walking, running, climbing only using their legs and feet, using wing flapping to assist their hindlimbs during climbing, and flying using vigorous wing flapping (2). Unfortunately, the links between these forms of locomotion and the incidence of KBD are not well understood. However, exciting new research has revealed that modifying aviary design to include ramps from the ground to elevated tiers and between tiers has the potential to reduce the frequency of falls or collisions and resulting KBD (3). We are only beginning to understand the capacity of domestic chickens to move over such ramps (4) and more fully exploit three-dimensional space within aviaries (2, 5).

Moving up an incline, in comparison to level ground, requires greater physical effort and is more challenging for animals to control (6). The increased demands upon the system are proportional to the angle of inclination (7). Recent work has demonstrated domestic chickens walk to climb 40° inclines and use wing-assisted incline running [WAIR; (8)] or aerial ascent when moving up steeper slopes (4).

Climbing performance may vary with age of development. For terrestrial birds in the Galliformes (chickens, pheasants, and relatives), ontogenetic effects on climbing performance varies among species. This variation appears to be due to rates of leg and wing growth relative to total change in body mass. After the wings develop sufficiently to produce aerodynamic force, birds employ WAIR (8–10). When growth in total mass outpaces wing development as it does in brush turkeys (*Alectura lathami*), hatchlings outperform adults (11). In contrast, increasing aerodynamic capacity outpaces total growth in chukar partridge (*Alectoris chukar*), and climbing performance increases throughout ontogeny (8–10).

Only recently has research been directed toward understanding how animals adapt their locomotion to navigate complicated terrain. Birds appear to use visual cues and they emphasize safety when moving over uneven terrain (12). They may accomplish this using active neural control of leg mechanics. For example, pheasants (*Phasianus colchicus*) control their limb during placement to achieve stability when walking or running over uneven terrain (13). As falls and collisions are at least partly responsible for KBD in chickens (3), we hypothesize that adaptive, anticipatory movements will improve safety for chickens navigating ramps within aviaries, but we do not presently know whether chickens respond to visual cues about incline and adjust their locomotor behavior. Furthermore, nothing is known about how developmental age affects adaptive locomotion in birds, and beginning to address this gap in knowledge is one of our objectives herein.

As part of our overall goal of elucidating causes and solutions to reduce the frequency of KBD, we undertook the present study to test whether incline angle and developmental age affect adaptive locomotion in domestic chickens. Morphology changes during development, so we measured body mass and leg length to test for effects of these variables upon performance (14). We measured forces exerted by the birds *via* their leg and foot upon the ground to test for preincline, anticipatory behavior in domestic chickens. Because the challenge of climbing increases with incline angle, we expected that effective anticipation of incline would be associated with increased peak ground force production and duration of time that the foot is in contact with the ground and decreased variation in the center of pressure (COP) under the foot, which provides a measure of an individual’s control over balance (15). Higher vertical peak forces from the leg and foot increase climb velocity at the start of the ramp, reduced range in COP excursion and COP sway velocity indicating greater postural stability, and increased foot contact time permits active control of limb posture (13). We predicted such anticipatory behavior would vary with increasing age during development, as is the case for overall climb performance in related bird species (9, 16).

## Materials and Methods

### Study Animals and Housing

Twenty female domestic chickens (*Gallus gallus domesticus*) comprising four strains (Lohmann Brown, Lohmann LSL, Dekalb White, and Hyline Brown) aged 17 weeks were divided into four home pens with five birds of a given strain per pen. The pens (183 L × 244 W × 290 H cm) contained 5 cm wood shavings on the floor, two elevated platforms on each side (122 L × 31 W at 70 and 160 cm above ground) and a wooden ramp (width = 15 cm) and ladder (width = 15 cm) connecting the elevated platforms at a 45° angle. An elevated, spring mounted (to simulate natural compliance in a tree-branch) perch spanning the width of the pen, was mounted 14 cm above the highest platform toward the rear end of the pen, with the two halves of the perch different in diameter (2.5 vs. 5 cm Ø). The pens also featured automatic drinkers, nest boxes, and a feeder. Room temperature averaged 21°C. The light cycle was 14:10 h light:dark, with a 30-min dawn and dusk. The light intensity was 50 lux.

### Ramp Apparatus

**Figure 1:**
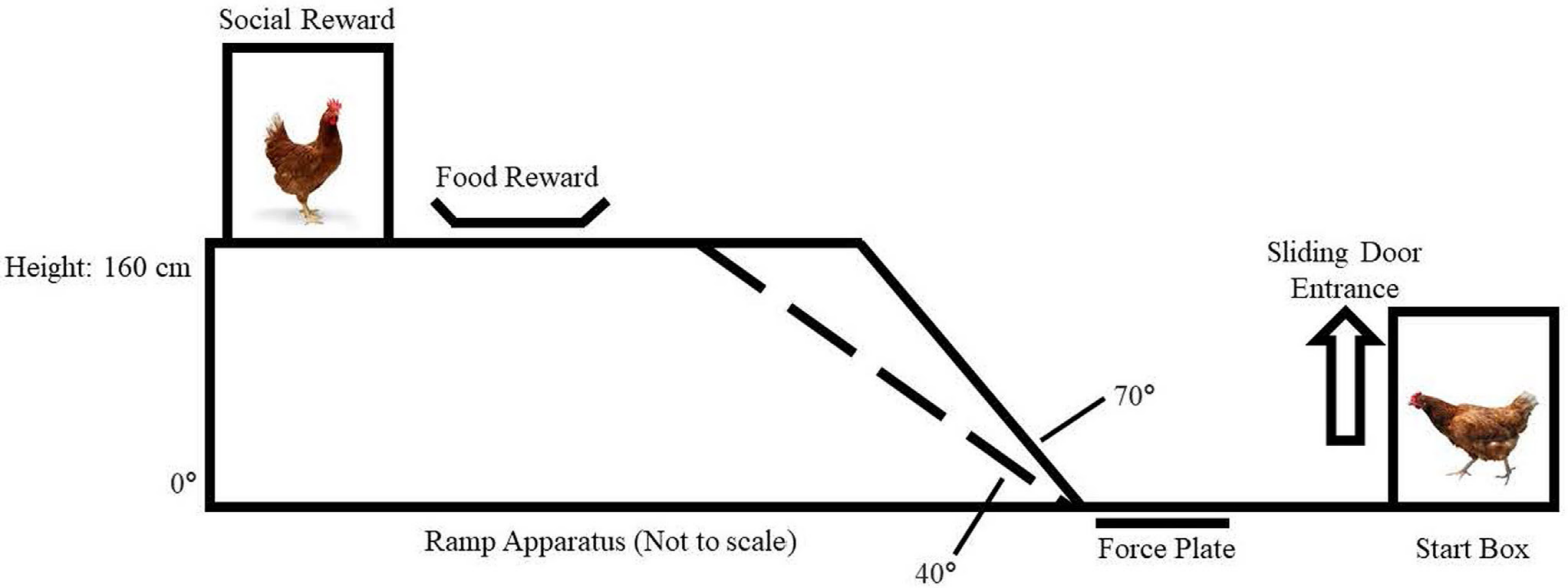
Ramp apparatus.

We constructed a ramp apparatus (Figure 1) with an adjustable ramp incline in a test room. The walkway consisted of a wooden start box (51 L × 31 W × 48 H cm) with a sliding door (47 H × 15 W cm) that could be lifted from outside of the test room. The start box opened to a level walkway (37 L × 15 W cm) made of a force plate (15 L × 15 W cm) with sandpaper (60-grit) applied to its surface for improved traction.

The ramp incline was attached with hinges to three interchangeable ramps that were horizontal (slope of 0°) or inclined (+40° and +70°). The ramp itself was 15-cm wide without directly adjacent walls. The ramps were covered with 2.5 cm^2^ wire mesh grid to improve grip and rested on an elevated platform (41 L × 15 W × 160 H cm) that was also covered with sandpaper (60-grit). In separate experiments (4), we confirmed that the ramp being 15-cm wide did not inhibit chickens from using their wings for balance or flapping them in WAIR; the same ramp widths are used in comparable studies of wild-type Gallifomes (9, 10, 16).

### Force Plate

Our custom-made force plate (Bertec Corporation, Columbus, OH, USA) had a square platform 15 cm on a side and had a resonant frequency of 200 Hz. We used an internal digital preamplifier (Bertec AM6500). The 16-bit digital signal was transferred to computer *via* RS-485. Bertec Digital Acquire software (version 4.0.11) was used to obtain the force plate data recorded at a frequency of 1,000 Hz. It recorded sic channels of internally calibrated data including three axes of force [vertical = *z*, lateral = *y*, and fore-aft = *x*, in Newtons (N), and the turning moments (torque) about each of these three axes (in Newton × meters, Nm)]. A Loess model (local regression smoothing process) in R (version 3.1.0), package: stats (17) was used to smooth the raw data following the methods of Cleveland et al. (18). Our variables for subsequent analysis included onset and offset time of ground reaction force (*t*_1_ and *t*_2_; s), peak vertical force (F*z*, N), range of excursion of the COP about the fore-aft (*x*) and lateral (*y*) axes (meters; COP*x* and COP*y*, respectively). We computed COP velocity about the *x* and *y* axes (m/s; V*x* and V*y*) using change in COP*x* and COP*y* as a function of time (i.e., *t*_2_ − *t*_1_).

### Training and Testing

Birds had experience [as part of a different study; (4)] with the ramp apparatus over a series of sessions during weeks 8, 9, 11, 13, and 15 on walkway inclines of 0° (horizontal control) +30°, +40°, +50°, +60°, and +70° angles reaching a platform height of 160 cm.

Birds were tested in a randomized order on/ramp inclines that were horizontal (slope of 0°) and inclined at +40° and +70° when they were 17, 21, 26, 31, and 36 weeks of age, with the goal of obtaining one successful trial per testing session for a given bird. Birds were placed in the start box for a period of 3 s until the start door was lifted by the experimenter. The bird had 60 s (determined from pilot study) to walk out of the start box, step on the force plate, and climb up the ramp. Birds were provided with motivation to climb using five pen mates in a crate on the elevated platform in addition to providing a tablespoon of their commercial feed along with five raisins. Each bird was given a second attempt if she failed in the first trial, defined as refusing to move up the ramp, moving away from the ramp apparatus, reaching the 60 s maximum to ascend the incline, or failing to step on the force plate. Only one trial was subsequently analyzed. Only trials where the bird placed one entire foot on the force plate were used for quantitative analysis in this study. To avoid visually distracting the birds, the experimenters observed each test from outside the test room using a video camera (JVC GC-PX100BU HD Everio) remotely connected *via* an iPad^®^ (Apple, Inc.). The videos served to see the entire bird and to confirm placement of one or two feet on the force plate.

### Morphological Measurements

We measured the weight and limb dimensions of the birds to aid in interpretation of changes in locomotor performance according to age. Birds were weighed at the end of 17, 21, 26, 31, and 36 weeks of age. After weighing, birds were restrained such that they were lying on their left side against a gridded background in order to photograph them using a digital camera (JVC GC-PX100BU HD Everio). We used Image J (v1.30, National Institutes of Health, Bethesda, MD, USA) to measure tibiotarsus, tarsometatarsus, and total leg length of the right leg in these digital images. Summary morphological data are presented in Table 1.

**Table 1:** Morphological measures for laying hens.

Age	*N*	Weight (kg)	Right tibiotarsus	Right tarsometatarsus	Total leg
Weeks			Length (cm)	Length (cm)	Length (cm)
17	20	1.37 ± 0.10	9.4 ± 0.6	7.2 ± 0.6	16.6 ± 0.9
21	20	1.72 ± 0.13	9.5 ± 0.3	7.2 ± 0.2	16.8 ± 0.5
26	20	1.87 ± 0.17	9.7 ± 0.1	7.3 ± 0.7	17.0 ± 0.3
31	20	1.94 ± 0.18	9.9 ± 0.4	7.3 ± 0.4	17.2 ± 0.7
36	20	1.98 ± 0.17	10.0 ± 0.8	7.4 ± 0.6	17.4 ± 1.1

*Mean values ± SD are presented*.

### Statistical Analysis

Our statistical analysis was designed to test whether incline angle significantly affected the forces and timing of anticipatory behavior. In addition, we tested for effects of age and strain upon these forces and timing. We initially categorized step events according to whether the stance phase featured one or two feet in contact with the plate. To focus on single-leg forces, we limited our subsequent analysis to the tests where the birds placed one foot on the force plate. We tested for effects of incline upon locomotor kinetics using the generalized linear mixed model procedure (PROC GLIMMIX) in SAS version 9.4 [SAS Institute Inc., Cary, NC, USA, 2012; (19)]. Because each chicken was repeatedly tested at different ages and inclines, we conducted a repeated measures variance analysis for each variable. Age of the birds, incline and interaction (age × incline) were included as fixed effects. Strain of the birds was removed from analysis, as there were no significant strain effects. We also conducted analysis of variance for the tibiotarsus, tarsometatarsus, and total limb length, with age of the birds was included as the fixed effect. Results are presented as mean estimates ± SD; we consider statistically significant differences among means to be *P* < 0.05.

### Ethical Statement

This study was approved by the University of Guelph Animal Care Committee (Animal Utilization Protocol # 2501) before testing.

## Results

All birds stepped with one foot on the force plate for the horizontal (slope of 0°) and +40° ramp incline at each of the five (17, 21, 26, 31, and 36 weeks of age) weeks of testing. To prepare to ascend the +70° incline, 15–45% of the birds within a given age class stepped with two feet on the force plate. To prepare for aerial ascent using hindlimb thrust and wing flapping, the birds placed both of their feet on the force plate, directly in front of the ramp. Hereafter, we present data from one-foot force plate contacts, meaning our sample size for statistical analyses was limited to the same subset of birds that stepped with one foot on the platform regardless of incline (week 17: *N* = 11 birds; week 21: *N* = 7 birds; week 26: *N* = 13 birds; week 31: *N* = 10 birds; week 36: *N* = 10 birds). Summary statistics for force variables are presented in Table 2. The magnitude of the peak vertical GRFs (relative to bodyweight) showed a significant increase at the greatest angle (*F*_2, 37.55_ = 28.88, *P* < 0.0001). There were no significant differences between horizontal and +40° ramp inclines, whereas higher vertical peak GRFs were measured on +70° ramp inclines compared to +40° ramp incline (−1.31 ± 0.18, *t*_17.49_ = −7.32, *P* < 0.0001) and compared to the horizontal ramp (−1.34 ± 0.18, *t*_17.08_ = −7.58, *P* < 0.0001).

**Table 2:** Mean values (±SD) of locomotor forces one step before the transition to climbing.

Age	Incline	Peak vertical force relative to body weight (Fz)	Contact time	Fore-aft excursion of center of pressure under foot (COP*x*)	Lateral excursion of center of pressure under foot (COP*y*)	Lateral velocity of the center of pressure (COP)	Fore-aft velocity of the COP
Weeks	Degrees (°)	Dimensionless	s	m	m	m/s	m/s
17	0	1.07 ± 0.20^A^	3.78 ± 5.52^A^	0.0007 ± 0.0011	0.0002 ± 0.0005	−0.024 ± 0.148	−0.044 ± 0.114
	+40	1.04 ± 0.19^A^	12.09 ± 23.39^B^	0.0007 ± 0.0009	0.0003 ± 0.0004	−0.016 ± 0.066	−0.020 ± 0.750
	+70	2.24 ± 0.45^B^	31.17 ± 21.21^C^	0.002 ± 0.003	0.0010 ± 0.0015	−0.014 ± 0.061	−0.011 ± 0.044
21	0	1.02 ± 0.19^A^	2.86 ± 6.75^A^	0.022 ± 0.092	0.014 ± 0.058	0.369 ± 0.990	0.236 ± 0.769
	+40	0.98 ± 0.29^A^	7.49 ± 16.95^B^	0.001 ± 0.004	0.0004 ± 0.0001	0.037 ± 0.155	−0.008 ± 0.116
	+70	2.86 ± 1.36^B^	13.77 ± 25.43^C^	0.006 ± 0.011	0.003 ± 0.006	−0.039 ± 0.087	−0.037 ± 0.061
26	0	1.07 ± 0.23^A^	3.30 ± 6.76^A^	0.001 ± 0.001	0.0003 ± 0.0002	0.047 ± 0.366	0.001 ± 0.209
	+40	1.16 ± 0.39^A^	6.72 ± 12.75^B^	0.0007 ± 0.001	0.0001 ± 0.0003	0.087 ± 0.138	0.006 ± 0.027
	+70	2.81 ± 1.09^B^	10.09 ± 12.57^C^	0.002 ± 0.003	0.001 ± 0.002	−0.029 ± 0.060	−0.037 ± 0.054
31	0	1.08 ± 0.17^A^	5.16 ± 11.12^A^	0.062 ± 0.273	0.052 ± 0.231	−0.052 ± 0.667	−0.122 ± 0.638
	+40	1.05 ± 0.39^A^	6.50 ± 12.41^B^	0.075 ± 0.321	0.026 ± 0.114	0.437 ± 1.568	0.243 ± 0.996
	+70	2.81 ± 1.51^B^	11.00 ± 9.91^C^	0.017 ± 0.029	0.012 ± 0.017	0.122 ± 0.586	0.064 ± 0.428
36	0	1.13 ± 0.34^A^	1.30 ± 1.15^A^	0.008 ± 0.023	0.004 ± 0.012	0.158 ± 0.256	0.059 ± 0.173
	+40	1.07 ± 0.45^A^	10.72 ± 17.39^B^	0.005 ± 0.013	0.002 ± 0.006	−0.078 ± 0.197	−0.034 ± 0.119
	+70	2.39 ± 1.12^B^	25.64 ± 24.44^C^	0.008 ± 0.024	0.004 ± 0.012	0.017 ± 0.030	0.002 ± 0.013

*Superscript letters indicate statistically significant differences among means (*P* ≤ 0.05)*.

Contact time depended on the angle of the incline (*F*_2, 41.28_ = 10.73, *P* = 0.0002). There were significantly longer foot contact times for birds transferring to the +70° ramp inclines compared to when they were transferring to +40° ramp incline (−11.27 ± 4.95, *t*_26.36_ = −2.27, *P* = 0.031) and to the horizontal ramp (−17.12 ± 4.60, *t*_19.81_ = −3.72, *P* = 0.001). Foot contact times were also significantly longer on the +40° ramp inclines in comparison to the horizontal ramp (−5.85 ± 1.99, *t*_78.28_ = −2.95, *P* = 0.004).

We did not observe significant differences in COP excursion, COP as an output of a control process to maintain postural control, and COP sway velocity about the *x*- (back/forth) and *y*- (left/right) axes in the last step before transitioning between the level and the inclined surface condition.

There was no evidence of a significant relationship between age or hindlimb dimensions and kinematic force plate data [vertical peak GRF (relative to body weight), ground contact time, excursion of COP, COP sway velocity] when transitioning from level ground to an incline. Tibiotarsus length of domestic chickens depended on their age (*F*_4, 58_ = 3.41, *P* = 0.014). At 36 weeks of age, birds had a significantly longer tibiotarsus length compared to week 17 (−0.65 ± 0.19, *t*_58_ = −3.52, *P* = 0.0009) and week 21 (−0.52 ± 0.24, *t*_58_ = −2.19, *P* = 0.032) (Table 1). There was no age effect for tarsometatarsus length.

## Discussion

Our results provide novel insight into the capacity for adaptive locomotion in domestic chickens. Chickens anticipated ascending an approaching incline as revealed by changes in GRFs and ground contact time. The most significant changes to a preincline step occurred only at the steepest incline angle we measured (+70°). Birds maximized their incline performance by increasing GRF accelerating upward and forward much as is the case during takeoff from a perch (20). There was no significant difference in peak vertical GRFs between level walking and preparing to walk the +40° incline. Similarly, domestic chickens do not change their mode of locomotion from walking to WAIR between horizontal and +40° inclines (4). Although ground contact time was 2.7 times longer one step before the transition to the +40° incline, it was 5.8 times longer before the transition to the +70° incline.

During most of the interval of ground contact, the weight exerted on the force plate was equal to body weight (both feet) or half body weight (one foot). This indicates a stance phase rather than a launch as might be observed in takeoff from a perch (20). Increased time for force application could allow a bird to generate more vertical velocity of its center of mass during the transition to the first step on the ramp because the velocity of the center of mass is ground contact impulse (i.e., force × time) divided by mass (20). However, our observations of long-duration contact times (Table 2) suggest the hens were engaged in decision making to prepare for the climb or perhaps, reacting with fear due to relative novelty of the situation. A minimum contact time of 1.3 s seems to indicate a slow walk, and the maximum contact time of 31 s might counts as a stand, indicating that birds paused and halted their forward momentum. Individuals exhibited great variation in force plate contact time as indicated by SDs about the means (Table 2).

Although leg length and body mass increased during the interval of 17–36 weeks (Table 1), we observed no differences in the mechanics of the preparatory step. It is likely that extending our experimental design to include younger birds would reveal age and size effects as have been shown for climbing in related Galliform species (9, 16). Future efforts to understand locomotion in relation to ramps in housing systems would do well to include younger age classes of hens.

There were no significant differences in COP excursions as a measure of postural stability or COP sway velocity between level walking and preparing to walk the +40° incline or +70° incline. From studies of developing humans (21), we expected an increase in postural stability and sway as inclines became steeper and a decrease in postural stability sway as the birds aged. The lack of significant effects of incline upon COP excursion and velocity is encouraging for the use of ramps in housing systems, as it indicates that domestic hens are capable of adaptive locomotion similar to closely related wild species (13).

One potential limitation of our study is due to the design of housing for the chickens. Hens had access to a ramp set at +45° in their home pen. This ramp is relatively close to the ramp set at +40° during the experiments, so the birds were likely more familiar with moderate incline climbing compared to climbing at +70°. Their relatively greater familiarity with moderate inclines may explain their significant modulation of force and contact time in preparation for climbing at +70°. This should be taken into account when interpreting our results.

Overall, our results revealed that domestic chickens have the capacity for adaptive locomotion in anticipating different grades of incline and that inclines of <40° require less modulation of kinetics than at steeper angles. Thus, we encourage the addition of relatively shallow ramps to rearing aviaries if such features are consistently shown to reduce the frequency of KBD (3). Peak vertical GRFs and contact time scale accordingly with the angle of the incline, which provides novel insight into the mechanics of anticipatory locomotor performance in layer hens.

## Ethics Statement

This study was approved by the University of Guelph Animal Care Committee (Animal Utilization Protocol # 2501) before testing.

## Author Contributions

CL collected data, participated in data analysis, and drafted the manuscript; BT participated in the design of the study, participated in data analysis, and drafted the manuscript, BS carried out statistical analysis and contributed analysis tools; AH-M conceived and designed the study, coordinated the study, participated in data analysis, and helped draft the manuscript. All authors gave final approval for publication.

## Conflict of Interest Statement

The authors declare that the research was conducted in the absence of any commercial or financial relationships that could be construed as a potential conflict of interest.
